# Changes in Microbial Energy Metabolism Measured by Nanocalorimetry during Growth Phase Transitions

**DOI:** 10.3389/fmicb.2018.00109

**Published:** 2018-02-01

**Authors:** Alberto Robador, Douglas E. LaRowe, Steven E. Finkel, Jan P. Amend, Kenneth H. Nealson

**Affiliations:** ^1^Center for Dark Energy Biosphere Investigations, University of Southern California, Los Angeles, CA, United States; ^2^Department of Earth Sciences, University of Southern California, Los Angeles, CA, United States; ^3^Molecular and Computational Biology Section, Department of Biological Sciences, University of Southern California, Los Angeles, CA, United States; ^4^Marine and Environmental Biology Section, Department of Biological Sciences, University of Southern California, Los Angeles, CA, United States

**Keywords:** calorimetry, *Shewanella oneidensis*, energy metabolism, microbial growth, thermodynamics

## Abstract

Calorimetric measurements of the change in heat due to microbial metabolic activity convey information about the kinetics, as well as the thermodynamics, of all chemical reactions taking place in a cell. Calorimetric measurements of heat production made on bacterial cultures have recorded the energy yields of all co-occurring microbial metabolic reactions, but this is a complex, composite signal that is difficult to interpret. Here we show that nanocalorimetry can be used in combination with enumeration of viable cell counts, oxygen consumption rates, cellular protein content, and thermodynamic calculations to assess catabolic rates of an isolate of *Shewanella oneidensis* MR-1 and infer what fraction of the chemical energy is assimilated by the culture into biomass and what fraction is dissipated in the form of heat under different limiting conditions. In particular, our results demonstrate that catabolic rates are not necessarily coupled to rates of cell division, but rather, to physiological rearrangements of *S. oneidensis* MR-1 upon growth phase transitions. In addition, we conclude that the heat released by growing microorganisms can be measured in order to understand the physiochemical nature of the energy transformation and dissipation associated with microbial metabolic activity in conditions approaching those found in natural systems.

## Introduction

Our goal was to use calorimetry to investigate how rates of catabolism scale with anabolism during the first three phases of the microbial growth cycle under laboratory conditions: lag phase, exponential or logarithmic phase, and stationary phase (Finkel, 2006). Cells undergo specific physiological responses during each of these phases, specific to the needs of the microbial population. During lag phase, immediately after cells are introduced into fresh medium, the previously quiescent cells begin to “retool” the macromolecular machinery required for cells to reach their maximum growth potential during the next phase. During exponential or logarithmic phase, cells will double with a generation time that is determined by the quantity and quality of available nutrients and energy sources. As the nutrients in the medium start to be depleted and metabolic waste products accumulate, cells transition into the third phase of the life cycle, stationary phase, where the apparent increase in biomass ceases. Although calorimetry has been used in conjunction with traditional physiological growth experiments to constrain the functional relationship between microbial activity and the physiological state of individual populations (Winkelmann et al., 2004; Schubert et al., 2007), little is known about how microorganisms couple catabolism to anabolism under different limiting culture conditions. The work presented here is based on the hypothesis that nanocalorimetric measurements of the relative total change in enthalpy of all of the reactions catalyzed by growing microorganisms, here referred to as metabolic heat, can be used to understand how microorganisms partition energy during growth and while under different substrate limitations. Here, we test this hypothesis experimentally.

## Materials and Methods

### Bacterial Strain and Growth Media

Stock batch cultures of *Shewanella oneidensis* MR-1 were routinely grown overnight aerobically at 30°C in 150-ml flasks containing 50 ml of Luria-Bertani (LB) broth, Miller (Difco) using an orbital incubator at 200 rpm. Chemostat cultures and batch cultures for calorimetry were incubated initially aerobically using a modified version of minimal growth medium with the following composition: 50 mM piperazine-*N,N*′-*bis*(2-ethanesulfonic acid) (PIPES), 7.5 mM NaOH, 28.04 mM NH_4_Cl, 1.34 mM KCl, 4.35 mM NaH_2_PO_4_.H_2_O, and 10 ml each of 100x vitamin solution, 100x mineral solution, and 100x amino acid solution (Kostka and Nealson, 1998). The medium was supplemented with 18 mM D,L-Lactate, which serves both as the energy and carbon source.

### Chemostat Cultivation

A 3-L New Brunswick Bioflow^®^/CelliGen^SM^ 115 reactor (New Brunswick Scientific, Edison, NJ, United States) operated aerobically at a 1-L working volume was used to grow chemostat cultures of *S. oneidensis* MR-1 at 30°C. An overflow system was used to maintain culture volume. Gas flow and agitation rates were therefore kept at 3.5 L/min and 300 rpm, respectively, and dissolved oxygen was maintained at 60% of air saturation by automatically changing the ratio of N_2_ and air in the gas mixture. pH was maintained at 7.0 by using a pH meter connected to an electrode and a pump to add sterile acid or alkali. The reactor was inoculated with 1 ml of overnight culture grown in LB and maintained in batch mode until late logarithmic phase (∼1 × 10^9^ CFU/ml). Continuous culture was initiated by pumping medium of the same composition at a dilution rate of 0.05 h^-1^. Carbon-, nitrogen-, or phosphorus-limiting growth conditions were achieved by decreasing the concentration of D,L-Lactate to 1 mM, NH_4_Cl to 0.1 mM, or NaH_2_PO_4_.H_2_O to 0.1 mM, respectively. Limiting growth conditions were inferred empirically by observing the increase in biomass yield upon increases in substrate concentrations at higher dilution rates, but not upon increases of dissolved oxygen in the medium (Kuenen, 2009) (data not shown).

### Measuring the Heat of Microbial Reactions with Calorimetry

Calorimetry experiments were initiated from either stock LB batch cultures of ∼10^9^ cells (for oxygen-limiting growth experiments) or chemostat cultures of ∼10^6^ cells (for carbon-, nitrogen-, or phosphorus-limiting growth experiments). Next, 1 ml each was serially diluted with modified M1 medium (amended with 18 mM D,L-Lactate) to a final concentration of ∼250 cells ml^-1^. Then, 4.2 ml each were transferred to calorimetric borosilicate ampules (cleaned and then combusted at 480°C for 6 h) allowing only 50 μl of headspace. In order to avoid the introduction of additional oxygen in the headspace calorimetry ampules were sealed with butyl rubber stoppers in a strict anaerobic atmosphere (<5 ppm oxygen and 5% hydrogen gas mix) using an anaerobic chamber (COY Laboratory Products, Inc., Grass Lake, MI, United States). Isothermal measurements of metabolic heat rates during the incubation of *S. oneidensis* MR-1 were performed in triplicate using a thermal activity monitor model TAM III equipped with a nanocalorimeter (TA Instruments, Lindon, UT, United States). The TAM III in combination with the nanocalorimeter offers extremely high sensitivity (>2.5 nW ml^-1^).

### Oxygen Uptake Analysis, Cells Enumeration, and Cell Size Measurements

Cellular oxygen uptake, number and size of growing cells of *S. oneidensis* MR-1 in calorimetric batch cultures was studied in parallel triplicates using a New Brunswick Bioflow^®^/CelliGen^SM^ 115 reactor (New Brunswick Scientific, Edison, NJ, United States) operated aerobically in batch mode and then sealed by layering 50 ml of white light mineral oil (Mallinckrodt, St. Louis, MO, United States) over the culture with a Clark-type oxygen microsensor (Unisense, Denmark) immersed in the culture reactor. The oxygen concentration in the reactor was logged every 5–10 s with the New Brunswick BioCommand^®^ supervisory computer software (New Brunswick Scientific, Edison, NJ, United States).

Total viable cell counts were determined at intervals of 2 h using an improved drop plate method (Herigstad et al., 2001). In short, 1 ml of the batch culture was serially diluted in sterile medium to a final countable dilution of 3–30 colonies per 10 μl drop of sample dispensed. The colony-forming units (CFUs) were counted over 10 drops at the countable dilution. Finally, the total count was scaled up and the viable cell counts were expressed as total CFUs in culture.

Quantitation of cell size of growing MR-1 cells was determined from microscopy images of fluorescently labeled cells as follows: 1 ml of the parallel batch culture was sampled and fixed in 0.2 μm filtered formalin [37–39% (wt/vol) formaldehyde solution] overnight at 4°C. Samples were then filtered onto polycarbonate membrane filters (type, GTBP; pore size, 0.2 μm; diameter, 2.5 mm; Sartorius, Göttingen, Germany) and stained with SYBR^®^ Green I (1:400 dilution from stock solution; Life Technologies, Carlsbad, CA, United States). Filters were mounted onto microscope glass slides with 0.1% (vol/vol) *p-*phenylenediamine anti-fade mounting medium and visualized using a Nikon Eclipse Ti-E inverted microscope (Nikon, Tokyo, Japan) equipped with a drift correction unit (Nikon Perfect Focus System) for maintaining focus at the coverslip-filter interface during imaging. Fluorescence imaging of SYBR Green I was done in the FITC (Nikon filter set B-2E/C). The scientific image analysis and visualization software program *DAIME* (Daims et al., 2006) was used to manually classify individual cells according to their measured caliper length and width. Rod-shaped MR-1 cells were assumed to be hemisphere-capped cylinders with radius *r* and height *h* (note: width/2 = *r* and length – 2*r* = *h*), and the cell volume was calculated by the equation *V* = 4/3(π*r*^3^) + π*r*^2^*h*. This is the volume of a sphere (two hemispheres) added to the volume of a cylinder.

### Cellular Protein Content

4 mL of stock culture samples (∼1 × 10^9^ CFU/ml) were used to determine the total protein content using the NanoOrange^®^ Protein Quantitation Kit (Molecular Probes, Inc., Eugene, OR, United States) following the manufacturer’s instructions. The average cell protein content was determined as a ratio of total protein content (ppm protein) to the cell concentration (CFU/mL). Cellular protein content of calorimetric batch cultures in lag and exponential growth phase were below detection limit (10 ng/ml) and therefore not reported here.

### Thermodynamic Calculations

The enthalpy of reaction for the full and partial oxidation of lactate by oxygen at 30°C and 1 bar was calculated for the following two reactions using the revised-HKF equations of state (Helgeson, 1981; Tanger and Helgeson, 1988; Shock et al., 1992), the SUPCRT92 software package (Johnson et al., 1992), and thermodynamic data taken from a number of sources (Shock, 1988; Shock et al., 1989; Shock and Helgeson, 1990; Sverjensky et al., 1997; Schulte et al., 2001):

(1) $$\begin{array}{c} {\text{C}}_{\text{3}}{\text{H}}_{\text{5}}{\text{O}}^{3-}(\text{Lactate})+3{\text{O}}_{2}+{\text{H}}^{+}\to 3{\text{CO}}_{\text{2}}{\text{+3H}}_{\text{2}}\text{O}\\ \Delta \;H_{r}^{{}^{\circ}}\;\text{at}\;{\text{30}}^{{}^{\circ}}\text{C}\;\text{and}\;\text{1}\;\text{bar}\;\text{=}\;\text{-458},\text{584}\;\text{J}\;{\left(\text{mol}\;{\text{O}}_{\text{2}}\right)}^{\text{-}}{}^{\text{1}} \end{array}$$

(2) $$\begin{array}{c} {\text{C}}_{\text{3}}{\text{H}}_{\text{5}}{\text{O}}^{3-}(\text{Lactate})+{\text{O}}_{2}\to {\text{CO}}_{\text{2}}{\text{+C}}_{2}{\text{H}}_{\text{2}}{\text{O}}^{2-}(\text{Acetate})+{\text{H}}_{\text{2}}\text{O}\\ \Delta \;H_{r}{}^{{}^{\circ}}\;\text{at}{\text{30}}^{{}^{\circ}}\text{C}\;\text{and}\;\text{1bar}\;\text{=}\;\text{-487},\text{278}\;\text{J}\;{\left(\text{mol}\;{\text{O}}_{\text{2}}\right)}^{\text{-}}{}^{\text{1}} \end{array}$$

The solubility of O_2_ in water at 30°C, and at a partial pressure of 0.21 bar, is 2.45 × 10^-4^ mol l^-1^. Since the modified M1 medium has an oxygen saturation of 89% of that in water (218 μM conc.) 4.2 ml of solution has 9.16 × 10^-7^ mol of O_2_.

## Results and Discussion

We incubated *S. oneidensis* MR-1 wild-type (Myers and Nealson, 1988) populations in a nanocalorimeter under a number of conditions, limiting the availability of either oxygen, carbon, nitrogen, or phosphorus. This γ-proteobacterium is an ideal model organism for (eco)physiological studies because it is globally distributed, can grow rapidly in the laboratory, and has been genomically and physiologically well-characterized (Hau and Gralnick, 2007). In each experiment described below, *S. oneidensis* MR-1 was incubated at 30°C, provided with oxygen and lactate as the source of energy and carbon under a variety of substrate-limited conditions in order to record the evolving metabolic heat as a function of time. The nanocalorimeter provides a continuous and sensitive measure of the flow of this heat out of a culture vial relative to a reference vial of the same heat capacity, consisting of filtered sterile growth medium. Thermodynamically, the heat measured represents the relative total change in enthalpy of the culture community. Thus, the heat change measured is due to the sum of all of the enthalpies of all of the reactions catalyzed by the microorganisms inside the culture vial (Kemp, 2000). Furthermore, if the enthalpy of a reaction under a particular condition is known, then this can be compared to the heat being measured by the calorimeter.

**FIGURE 1:**
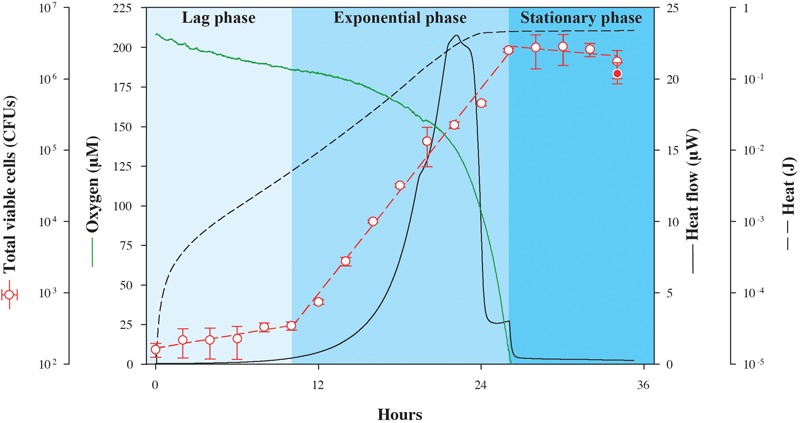
Nanocalorimetric analysis of *Shewanella oneidensis* MR-1 growth. Heat flow (solid black line) of *S. oneidensis* MR-1 growing on lactate (carbon source and electron donor) at pH 7 and 30°C under O_2_ limitation is shown. Total heat evolved during growth (dashed black line) was obtained by integrating the area delimited by the heat flow profile. The increase in total viable cells (red circles) was determined in parallel cultures using a drop plate method. For comparison, final cell growth in calorimetric vials is represented as the red filled circle, indicating that data from the calorimetric and parallel growth experiments were in good agreement. Oxygen concentration (green line) was also measured in a parallel culture outside the calorimeter. Corresponding heat and oxygen data of experimental triplicate measurements are shown in **Supplementary Figure S1**.

In the first experiment, excess lactate was made available to *S. oneidensis* MR-1 in an oxic setting while tracking the changes in heat flow. In identical, parallel experimental cultures incubated at the same temperature outside of the calorimeter, oxygen and biomass concentrations were also monitored so that the energy associated with catabolism could be related to physiological changes as a function of time (**Figure 1**). Because this is a closed system, the only O_2_ that is available is that which is present in the headspace and dissolved in the medium at the beginning of the experiment. As shown in **Figure 1**, after the lag phase (∼10 h), heat flow increases exponentially up to a maximum of 23.1 μW, and then falls sharply stepwise to a minimum of 0.6 μW at about 26 h, when oxygen had been completely consumed. Changes in viable cell counts followed the heat flow with a maximum growth rate of 0.24 h^-1^, peaking as the oxygen is ultimately consumed, but about 2 h after the maximum heat flow is detected. The total quantity of heat measured during growth, the integrated heat flow, was 0.475 J. Although *S. oneidensis* MR-1 can fully oxidize lactate to CO_2_ under fully aerobic conditions (Pinchuk et al., 2011), it only partially oxidizes lactate to acetate and CO_2_ when the amount of dissolved O_2_ approaches zero (Pinchuk et al., 2011). Therefore, it is possible that the heat signal shown in **Figure 1** is a composite of both of these oxidation reactions, since O_2_ becomes limiting over time. We calculate that if all of the O_2_ available in the experimental vial is used to completely oxidize lactate, then 0.42 J would be released, while if all of the consumed lactate is partially oxidized to acetate and CO_2_, 0.45 J would evolve. Although, it is difficult to determine the exact fate of lactate in this experiment, it is clear that the recorded heat signal is dominated by the enthalpy of lactate oxidation.

In order to quantify the amount of heat associated with anabolic processes during the experiment summarized in **Figure 1**, we estimated the heat associated with biomass synthesis using the heat of combustion for bacterial cells. Given that our incubation experiment yielded a total of 2.8 × 10^6^ cells with an estimated average cellular weight of 4 × 10^-7^ μg cell^-1^ [calculated from measured average cellular content values of 20 pg, which is consistent with previously published data (Saini and Wood, 2008), and assuming that protein content constitutes approximately 50% of the total cellular weight (Pinchuk et al., 2010)] and 22.9 J g^-1^ evolve from combusting *Escherichia coli* K-12 cells (Dermoun and Belaich, 1980), the enthalpy change accompanying anabolism results in -2.6 × 10^-5^ J (the sign change results from anabolism being the opposite of combustion). This relatively small amount of heat further demonstrates that most of the heat signal measured in the experiment arises from the catabolic reaction supporting the cells, lactate oxidation.

**FIGURE 2:**
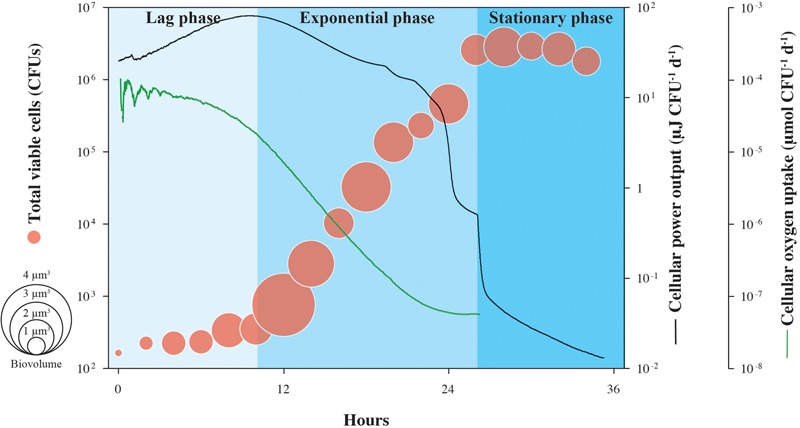
Cell-specific power output and oxygen uptake of growing *S. oneidensis* MR-1. Cellular heat yield and oxygen consumption rates for growth of *S. oneidensis* MR-1 were calculated from measured oxygen concentrations over time and normalized by the number of CFU shown in **Figure 1**. This estimate assumes equal contributions by all cells in the population to the heat yield and respiration rate. Circle size indicates the calculated cellular volume of growing *S. oneidensis* MR-1 cells.

Cell-specific oxygen consumption and heat generation rates for the same experiment shown in **Figure 1** are shown in **Figure 2**, in addition to average cell volumes. Oxygen demand is high during lag phase, peaking at 2.8 × 10^-3^ μmol O_2_ CFU^-1^ d^-1^, but falls by more than three orders-of-magnitude during the exponential growth phase, supporting the observation that respiration rates of *S. oneidensis* MR-1 cells are highly dynamic (Riedel et al., 2013). Similarly, cellular heat flow output reaches a maximum value of 15.7 μJ CFU^-1^ d^-1^ at the onset of the exponential growth phase, but by the end, drops by over three orders-of-magnitude. Through the transition from exponential phase to stationary phase, cellular heat flow decreases rapidly by nearly one order-of-magnitude. Cell volumes increase during lag phase and remain variable during exponential growth and stationary phase; the increase in cell numbers is not directly proportional to the increase in cell volumes.

The patterns shown in **Figure 2** could be explained by a range of physiological changes that *S. oneidensis* MR-1 cells are known to undergo during the various phases of their growth cycle. During lag phase, when the energy source (lactate) is found in excess in the medium and oxygen is plentiful, *S. oneidensis* MR-1 cells appear to generate more protein per cell (Saini and Wood, 2008), which could explain the increased cell size observed in our experiments. In this phase, the physiological retooling required for *S. oneidensis* MR-1 to begin exploiting newly replete nutrient conditions includes shifts in regulatory patterns of gene expression, metabolic pathways, internal accumulation and turnover of macromolecular compounds (Beg et al., 2012). All of these processes involve an increased demand of catabolic energy generally associated with rapid heat production (Russell, 2007). An open question with respect to lag phase physiology is whether the majority of these “building blocks” of the macromolecular cellular machinery accumulate during lag phase or continue to be synthesized throughout log phase growth. The increasing oxygen consumption measured during lag phase (and thus the associated high heat dissipation) suggests that *S. oneidensis* MR-1 is preparing for optimal growth and that, during the subsequent exponential phase, little more catabolic energy is needed. Furthermore, under the initially replete culture conditions encountered at inoculation, the catabolic activity of *S. oneidensis* MR-1 cells can transiently exceed their growth capacity and metabolic reactions are known to operate in *S. oneidensis* MR-1 (Pinchuk et al., 2010) to dissipate this excess energy. These results in a rapid increase in heat production (Russell, 1986; Russell and Strobel, 1990) and could further contribute to the high heat dissipation rate observed in lag-phase cells. These metabolic reactions are associated with futile enzyme cycles and dissipation of membrane potential providing *S. oneidensis* MR-1 cells not only a means of protection from potentially toxic concentrations of metabolic intermediates (Qian and Beard, 2006), but also providing them with a competitive advantage in energy rich environments (Pinchuk et al., 2010). Similarly, the cell-specific heat effect marking the transition from late exponential phase into stationary phase could be attributed to physiological rearrangements as a reaction to decreasing oxygen concentration. Together, our data indicate that heat production in bacteria is extremely variable across different growth phases, showing that calorimetric analysis of heat production is specific to cellular functions underlying metabolism and not necessarily proportional to the number of active cells, as previously thought (Braissant et al., 2013).

**FIGURE 3:**
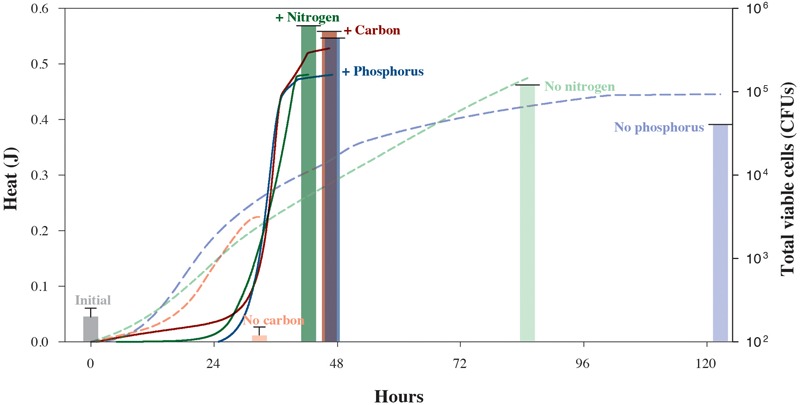
Heat yields and cell counts for *S. oneidensis* MR-1 cultures grown under carbon-, nitrogen-, or phosphorus-replete (solid lines and bold columns) and -limiting conditions (dashed lines and faded columns). Chemostat cultures enabled the reproducible growth of *S. oneidensis* MR-1 under limiting conditions. Cells were harvested from chemostats to monitor growth in the presence or absence of the respective limiting element in nanocalorimeter vial batch cultures. Solid and dashed lines refer to total heat evolved during the respective replete and depleted element conditions. Corresponding heat data of experimental triplicate measurements are shown in **Supplementary Figures S2–S4**. Total *S. oneidensis* MR-1 CFU abundances in calorimetric vessels are shown at the beginning and end of each experiment, with solid bars representing replete conditions and faded bars representing the limiting conditions. Error bars correspond to the calculated standard deviation of five replicate measurements.

We also carried out a series of isothermal nanocalorimetry experiments to gauge how *S. oneidensis* MR-1 cells use energy while growing under different limiting conditions. In these experiments, which are designed to approach common, natural environmental conditions, cells were incubated in chemostats with depletion of C, N, or P before being placed in the calorimeter. **Figure 3** illustrates the different impacts that nutrient-depleted media have on the rates of microbial growth. Based on the total amount of heat that evolved from the carbon-limited experiment, 0.23 J, less than half as much lactate was oxidized as compared to the carbon-replete experiment, 0.56 J. However, these catabolic rates did not translate into growth for the carbon-limited cells. The *S. oneidensis* MR-1 cells in the N- and P-limited experiments, however, produced nearly as much heat as the corresponding nutrient replete cultures, 0.48 and 0.45 J, respectively. Unlike the carbon-starved cells, however, these cultures do show some growth, a total of 5.0 × 10^5^ CFUs and 1.7 × 10^5^ CFUs, respectively. These results make sense in that carbon-energy-limited cultures of *S. oneidensis* MR-1 are typically characterized by a loss in cell mass due to oxidation of internal storage products for energy for cell maintenance (Tang et al., 2007), but N- and P-poor environments do not stop growth since this species can grow by utilizing N and P liberated from different sources, i.e., DNA (Pinchuk et al., 2008) and amino acids (Pinchuk et al., 2010). However, despite the lower growth yields of the C-, N-, and P-deficient experiments, when the total heat is normalized by the cell counts, maximum cell-specific heat rates were 203–527 μJ cell^-1^ d^-1^, over an order-of-magnitude higher than those observed while growing in nutrient-replete media. This indicates that an additional energy expenditure is necessary to mobilize the required nutrients, and reveals the physiological mechanisms underlying observed population shifts and selection of highly respiring cells under nutrient stress conditions (Riedel et al., 2013). The maintenance and expression of this reactivity necessary to respond rapidly to environmental fluctuations (Russell and Cook, 1995) leads to the notion that regulatory physiological processes in natural microbial communities are not necessarily arranged to achieve the maximum growth yield in the shortest possible timeframe (Tempest and Neijssel, 1978), but to survive under potentially harsh and variable conditions. Our experiments also demonstrate how nutrient-starved microbes possess the ability to sustain high rates of metabolic activity when amended with limiting substrates (Morono et al., 2011; Robador et al., 2015).

## Author Contributions

AR designed and performed the experiments, analyzed the data, and wrote the paper. AR performed and analyzed the calorimetry data. DL contributed to the analysis of calorimetry data, performed the thermodynamic calculations, and contributed to the preparation of the manuscript. SF contributed to the preparation of the manuscript. AR, JA, and KN conceived the study. JA and KN supported the study. All authors discussed the results and implications and commented on the manuscript at all stages.

## Conflict of Interest Statement

The authors declare that the research was conducted in the absence of any commercial or financial relationships that could be construed as a potential conflict of interest.

## Supplementary Material

FIGURE S1 **(A)** Triplicate heat-flow (solid gray lines) and oxygen concentrations (solid green lines) measurements during growth of *S. oneidensis* MR-1 under oxygen limiting conditions. **(B)** Total accumulated heat during growth. Data presented in **Figure 1** is highlighted in bold.

FIGURE S2 **(A)** Triplicate heat-flow measurements and **(B)** heat yields for *S. oneidensis* MR-1 cultures grown under carbon replete condition. Solid red lines refer to total heat evolved during the respective depleted element conditions. Data presented in **Figure 3** is highlighted in bold.

FIGURE S3 **(A)** Triplicate heat-flow measurements and **(B)** heat yields for *S. oneidensis* MR-1 cultures grown under nitrogen replete condition. Solid red lines refer to total heat evolved during the respective depleted element conditions. Data presented in **Figure 3** is highlighted in bold.

FIGURE S4 **(A)** Triplicate heat-flow measurements and **(B)** heat yields for *S. oneidensis* MR-1 cultures grown under phosphorus replete condition. Solid red lines refer to total heat evolved during the respective depleted element conditions. Data presented in **Figure 3** is highlighted in bold.

## References

[B1] Beg Q. K. Zampieri M. Klitgord N. Collins S. B. Altafini C. Serres M. H. (2012). Detection of transcriptional triggers in the dynamics of microbial growth: application to the respiratorily versatile bacterium *Shewanella oneidensis*. *Nucleic Acids Res.* 40 7132–7149. 10.1093/nar/gks467 22638572 PMC3424579

[B2] Braissant O. Bonkat G. Wirz D. Bachmann A. (2013). Microbial growth and isothermal microcalorimetry: growth models and their application to microcalorimetric data. *Thermochim. Acta* 555 64–71. 10.1016/j.tca.2012.12.005

[B3] Daims H. Lucker S. Wagner M. (2006). DAIME, a novel image analysis program for microbial ecology and biofilm research. *Environ. Microbiol.* 8 200–213. 10.1111/j.1462-2920.2005.00880.x 16423009

[B4] Dermoun Z. Belaich J. P. (1980). Microcalorimetry study of *Escherichia coli* aerobic growth: theoretical aspects of growth in succinic acid. *J. Bacteriol.* 143 742–746. 10.1007/BF00251852 7009563 PMC294354

[B5] Finkel S. E. (2006). Long-term survival during stationary phase: evolution and the GASP phenotype. *Nat. Rev. Microbiol.* 4 113–120. 10.1038/nrmicro1340 16415927

[B6] Hau H. H. Gralnick J. A. (2007). Ecology and biotechnology of the genus *Shewanella*. *Annu. Rev. Microbiol.* 61 237–258. 10.1146/annurev.micro.61.080706.093257 18035608

[B7] Helgeson H. C. (1981). Prediction of the thermodynamic properties of electrolytes at high-pressures and temperatures. *Phys. Chem. Earth* 1 133–177. 10.1016/0079-1946(81)90009-4

[B8] Herigstad B. Hamilton M. Heersink J. (2001). How to optimize the drop plate method for enumerating bacteria. *J. Microbiol. Methods* 44 121–129. 10.1016/s0167-7012(00)00241-4 11165341

[B9] Johnson J. W. Oelkers E. H. Helgeson H. C. (1992). SUPCRT92 – a software package for calculating the standard molal thermodynamic properties of minerals, gases, aqueous species, and reactions from 1 Bar and 0 degrees C to 1000 degrees C. *Comput. Geosci.* 18 899–947. 10.1016/0098-3004(92)90029-q

[B10] Kemp R. B. (2000). ‘Gie me ae spark o’ nature’s fire’ - An insight into cell physiology from calorimetry. *J. Therm. Anal. Calorim.* 60 831–843. 10.1023/a:1010199422705

[B11] Kostka J. Nealson K. H. (1998). “Isolation, cultivation and characterization of iron- and manganese-reducing bacteria,” in *Techniques in Microbial Ecology* eds Burlage R. S. Atlas R. Stahl D. Geesey G. Sayler G. (New York, NY: Oxford University Press) 58–78.

[B12] Kuenen J. G. (2009). “Continuous cultures (chemostats),” in *Encyclopedia of Microbiology* Vol. 6 ed. Schaechter M. (Oxford: Elsevier) 130–147. 10.1016/B978-012373944-5.00112-7

[B13] Morono Y. Terada T. Nishizawa M. Ito M. Hillion F. Takahata N. (2011). Carbon and nitrogen assimilation in deep subseafloor microbial cells. *Proc. Nat. Acad. Sci. U.S.A.* 108 18295–18300. 10.1073/pnas.1107763108 21987801 PMC3215001

[B14] Myers C. R. Nealson K. H. (1988). Bacterial manganese reduction and growth with manganese oxide as the sole electron-acceptor. *Science* 240 1319–1321. 10.1126/science.240.4857.1319 17815852

[B15] Pinchuk G. E. Ammons C. Culley D. E. Li S. M. McLean J. S. Romine M. F. (2008). Utilization of DNA as a sole source of phosphorus, carbon, and energy by *Shewanella* spp.: ecological and physiological implications for dissimilatory metal reduction. *Appl. Environ. Microbiol.* 74 1198–1208. 10.1128/AEM.02026-07 18156329 PMC2258558

[B16] Pinchuk G. E. Geydebrekht O. V. Hill E. A. Reed J. L. Konopka A. E. Beliaev A. S. (2011). Pyruvate and lactate metabolism by *Shewanella oneidensis MR*-1 under fermentation, oxygen limitation, and fumarate respiration conditions. *Appl. Environ. Microbiol.* 77 8234–8240. 10.1128/aem.05382-11 21965410 PMC3233039

[B17] Pinchuk G. E. Hill E. A. Geydebrekht O. V. De Ingeniis J. Zhang X. Osterman A. (2010). Constraint-based model of *Shewanella oneidensis* MR-1 metabolism: a tool for data analysis and hypothesis generation. *PLOS Comput. Biol.* 6:e1000822. 10.1371/journal.pcbi.1000822 20589080 PMC2891590

[B18] Qian H. Beard D. A. (2006). Metabolic futile cycles and their functions: a systems analysis of energy and control. *Mol. Syst. Biol.* 153 192–200. 10.1049/ip-syb:20050086 16986621

[B19] Riedel T. E. Berelson W. M. Nealson K. H. Finkel S. E. (2013). Oxygen consumption rates of bacteria under nutrient-limited conditions. *Appl. Environ. Microbiol.* 79 4921–4931. 10.1128/aem.00756-13 23770901 PMC3754705

[B20] Robador A. Jungbluth S. P. Larowe D. Bowers R. Rappe M. Amend J. P. (2015). Activity and phylogenetic diversity of sulfate-reducing microorganisms in low-temperature subsurface fluids within the upper oceanic crust. *Front. Microbiol.* 5:748. 10.3389/fmicb.2014.00748 25642212 PMC4295021

[B21] Russell J. B. (1986). Heat-production by ruminal bacteria in continuous culture and its relationship to maintenance energy. *J. Bacteriol.* 168 694–701. 10.1128/jb.168.2.694-701.1986 3782021 PMC213537

[B22] Russell J. B. (2007). The energy spilling reactions of bacteria and other organisms. *J. Mol. Microbiol. Biotechnol.* 13 1–11. 10.1159/000103591 17693707

[B23] Russell J. B. Cook G. M. (1995). Energetics of bacterial growth – Balance of anabolic and catabolic reactions. *Microbiol. Rev.* 59 48–62. 7708012 10.1128/mr.59.1.48-62.1995 PMC239354

[B24] Russell J. B. Strobel H. J. (1990). ATPase-dependent energy spilling by the ruminal bacterium, *Streptococcus bovis*. *Arch. Microbiol.* 153 378–383. 10.1007/bf00249009 2140038

[B25] Saini G. Wood B. D. (2008). Metabolic uncoupling of *Shewanella oneidensis* MR-1, under the influence of excess substrate and 3, 3’, 4’, 5-tetrachlorosalicylanilide (TCS). *Biotechnol. Bioeng.* 99 1352–1360. 10.1002/bit.21702 17972334

[B26] Schubert T. Breuer U. Harms H. Maskow T. (2007). Calorimetric bioprocess monitoring by small modifications to a standard bench-scale bioreactor. *J. Biotechnol.* 130 24–31. 10.1016/j.jbiotec.2007.02.013 17397956

[B27] Schulte M. D. Shock E. L. Wood R. H. (2001). The temperature dependence of the standard-state thermodynamic properties of aqueous nonelectrolytes. *Geochim. Cosmochim. Acta* 65 3919–3930. 10.1016/s0016-7037(01)00717-7

[B28] Shock E. L. (1988). Organic acid metastability in sedimentary basins. *Geology* 16 886–890. 10.1130/0091-76131988016<0886:oamisb<2.3.co;2

[B29] Shock E. L. Helgeson H. C. (1990). Calculation of the thermodynamic properties of aqueous species at high-pressures and temperatures – standard partial properties of organic species. *Geochim. Cosmochim. Acta* 54 915–945. 10.1016/0016-7037(90)90429-o

[B30] Shock E. L. Helgeson H. C. Sverjensky D. A. (1989). Calculation of the thermodynamic properties of aqueous species at high-pressures and temperatures – standard partial molal properties of inorganic neutral species. *Geochim. Cosmochim. Acta* 53 2157–2183. 10.1016/0016-7037(89)90341-4

[B31] Shock E. L. Oelkers E. H. Johnson J. W. Sverjensky D. A. Helgeson H. C. (1992). Calculation of the thermodynamic properties of aqueous species at high-pressures and temperatures –Effective electrostatic RADII, dissociation constants and standard partial molar properties to 1000 degrees C and 5 Kbar. *J. Chem. Soc. Faraday Trans.* 88 803–826. 10.1039/ft9928800803

[B32] Sverjensky D. A. Shock E. L. Helgeson H. C. (1997). Prediction of the thermodynamic properties of aqueous metal complexes to 1000 degrees C and 5 kb. *Geochim. Cosmochim. Acta* 61 1359–1412. 10.1016/s0016-7037(97)00009-4 11541435

[B33] Tang Y. J. J. Meadows A. L. Keasling J. D. (2007). A kinetic model describing *Shewanella oneidensis* MR-1 growth, substrate consumption, and product secretion. *Biotechnol. Bioeng.* 96 125–133. 10.1002/bit.21101 16865732

[B34] Tanger J. C. Helgeson H. C. (1988). Calculation of the thermodynamic properties of aqueous species at high-pressures and temperatures – revised equations of state for standard partial molal properties of ions and electrolytes. *Am. J. Sci.* 288 19–98. 10.2475/ajs.288.1.19

[B35] Tempest D. W. Neijssel O. M. (1978). “Eco-physiological aspects of microbial growth in aerobic nutrient-limited environments,” in *Advances in Microbial Ecology* Vol. 2 ed. Alexander M. (Boston, MA: Springer) 105–153.

[B36] Winkelmann M. Huttl R. Wolf G. (2004). Application of batch-calorimetry for the investigation of microbial activity. *Thermochim. Acta* 415 75–82. 10.1016/j.tca.2003.08.028

