# Supplementary figures and images for: Changes in Microbial Energy Metabolism Measured by Nanocalorimetry during Growth Phase Transitions
Source: Front Microbiol. 2018 Feb 1;9:109. doi: 10.3389/fmicb.2018.00109 (PMC5800293; doi:10.3389/fmicb.2018.00109)

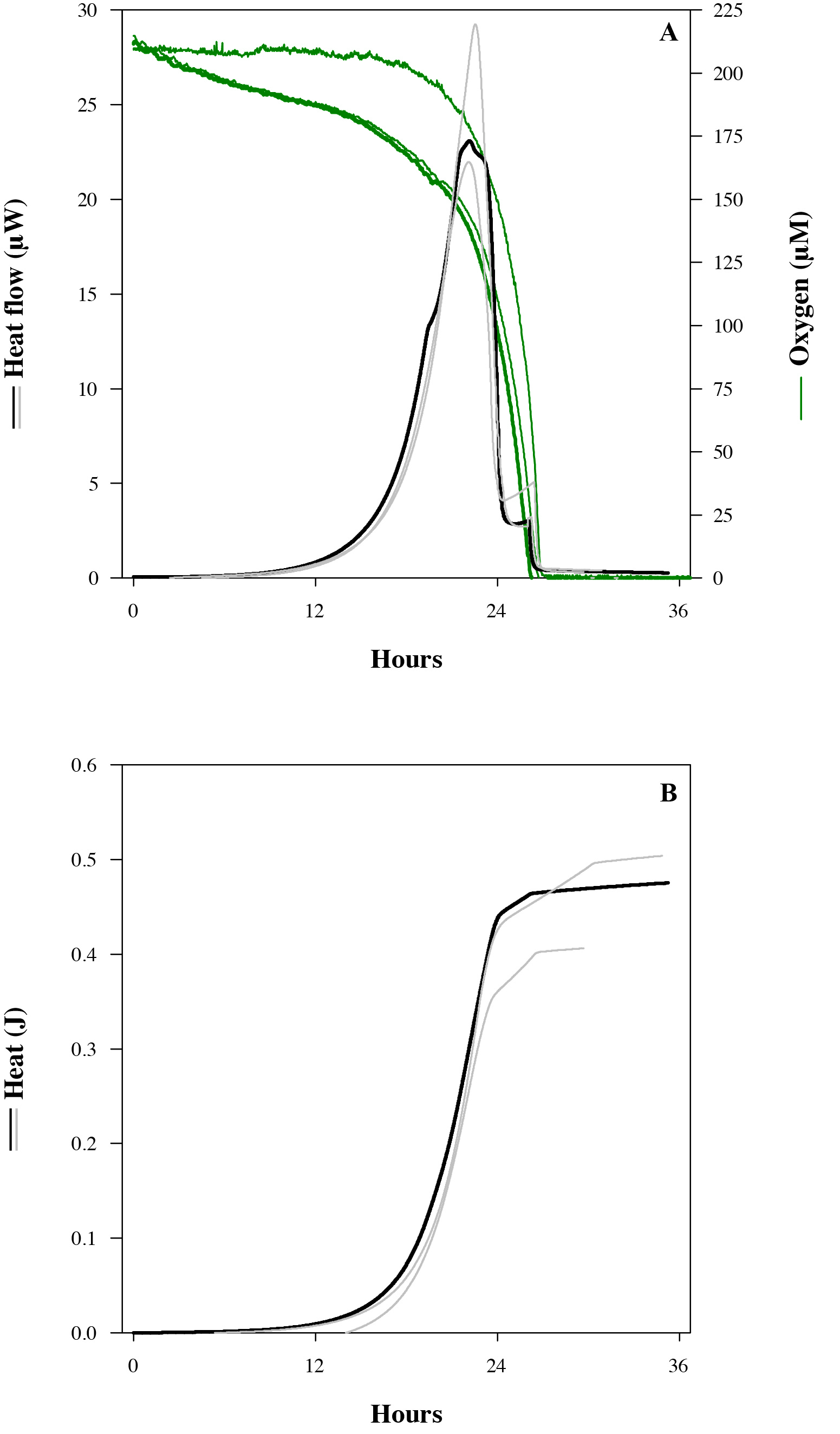

Supplement: FIGURE S1 — (A) Triplicate heat-flow (solid gray lines) and oxygen concentrations (solid green lines) measurements during growth of S. oneidensis MR-1 under oxygen limiting conditions. (B) Total accumulated heat during growth. Data presented in Figure 1 is highlighted in bold. [file Image_1.JPEG]

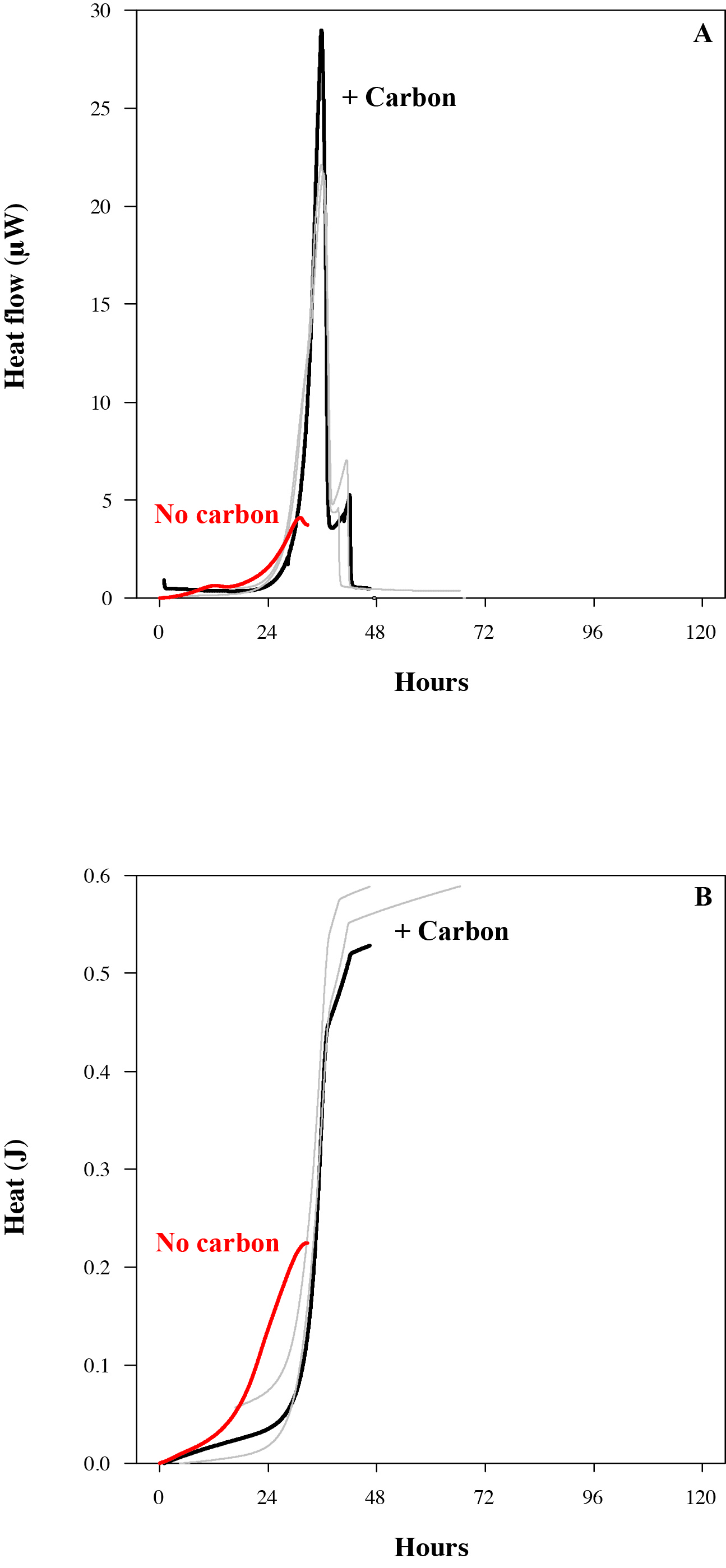

Supplement: FIGURE S2 — (A) Triplicate heat-flow measurements and (B) heat yields for S. oneidensis MR-1 cultures grown under carbon replete condition. Solid red lines refer to total heat evolved during the respective depleted element conditions. Data presented in Figure 3 is highlighted in bold. [file Image_2.JPEG]

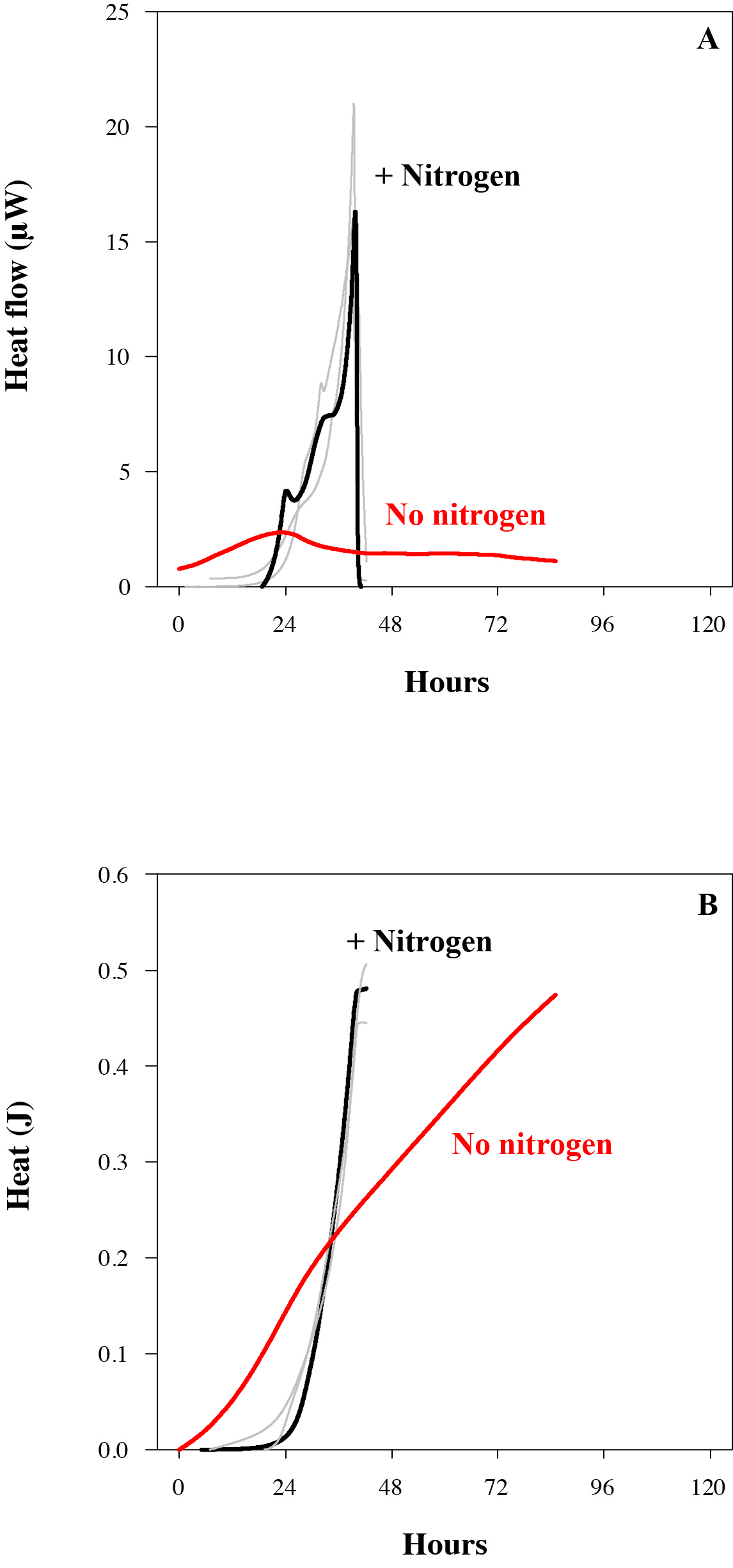

Supplement: FIGURE S3 — (A) Triplicate heat-flow measurements and (B) heat yields for S. oneidensis MR-1 cultures grown under nitrogen replete condition. Solid red lines refer to total heat evolved during the respective depleted element conditions. Data presented in Figure 3 is highlighted in bold. [file Image_3.JPEG]

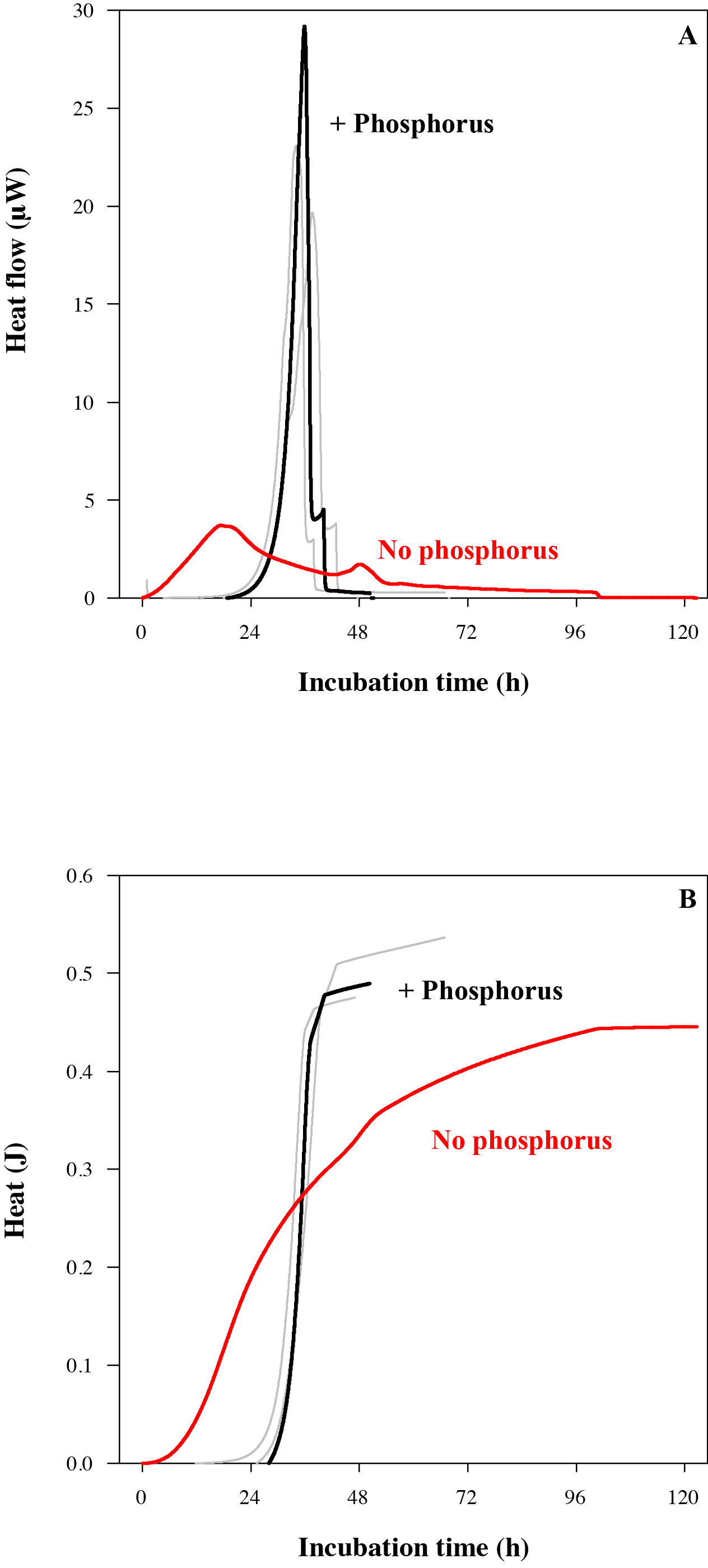

Supplement: FIGURE S4 — (A) Triplicate heat-flow measurements and (B) heat yields for S. oneidensis MR-1 cultures grown under phosphorus replete condition. Solid red lines refer to total heat evolved during the respective depleted element conditions. Data presented in Figure 3 is highlighted in bold. [file Image_4.JPEG]
